# Comparison of Graft Uptake in Perforated Eardrums with and without Myringosclerosis: A Prospective Case-control Study in a Tertiary Centre

**DOI:** 10.1055/s-0044-1791730

**Published:** 2025-01-31

**Authors:** Ayaz Rehman, Faizah Ashfah Latief Deva, Asef Ahmad Wani, Majid Masoodi, Bashir Malik, Owais Makhdoomi

**Affiliations:** 1 Department of Otorhinolaryngology, Head and Neck Surgery, SKIMS Medical College and Hospital, Srinagar, Jammu and Kashmir, India

**Keywords:** otitis media, tympanic membrane perforation, sclerosis, tympanoplasty, uptake, outcome

## Abstract

**Introduction** Various factors have been reported to affect the rates of success after tympanoplasty, among them, myringosclerosis. However, there are few studies focusing only on the effect of myringosclerotic plaque removal on tympanoplasty outcomes.

**Objective** To compare the outcome of tympanoplasty in perforated eardrums with and without myringosclerosis.

**Methods** The study included patients aged between 21 and 53 years diagnosed at the outpatient department with inactive mucosal chronic otitis media. The sample was divided into the case group, which included subjects with tympanic membrane perforation and myringosclerosis, and the control group, which included subjects with tympanic membrane perforation only, without myringosclerosis. We assessed the audiological findings of the patients before and after surgery, as well as hearing gain, graft uptake, and residual perforation/reperforation.

**Results** No significant relationships were observed involving the age or sex of the patient and the closure rate or hearing gain, neither between the location of the perforation and graft uptake. Graft uptake was higher in patients with perforation size < 50%. The graft uptake and hearing gain were higher in the case group.

**Conclusion** The removal of sclerotic plaques increases the surface of the raw area created by subepithelial excision of the myringosclerotic plaques, leading to a high rate of surgical success for the closure of tympanic membrane defects with coexisting myringosclerosis.

## Introduction

Chronic otitis media (COM) is defined as an inflammation of the middle ear and its cleft, consisting of the Eustachian tube, the tympanic cavity, and mastoid cells, with signs of infection lasting 3 months or longer. Chronic suppurative otitis media (CSOM) is characterized by persistent otorrhea from a perforated tympanic membrane (TM) for more than 3 months. The presence of a permanent tympanic perforation and otorrhoea differentiates CSOM from other forms of COM. 1 Perforation of the TM can be caused by trauma, Eustachian tube dysfunction, and diseases of the middle ear cleft, and it can result in conductive hearing loss, ranging from 30 to 60 dB. 2

The accepted protocol for the management of COM is surgical and the type of procedure is planned considering its pathological type. The surgical approach for tympanoplasty can be endaural, transmeatal, postauricular, or supramental. The temporalis muscle fascia and the perichondrium of the tragal cartilage are the most popular materials used as grafts, and they act as a scaffold for the growth of squamous epithelium from the TM remnant. The most common technique of grafting is underlay (medial to the TM remnant) and overlay (lateral the TM remnant). 3 4 The aims of tympanoplasty are the removal of the disease, the formation of a mucosal-lined middle ear cleft with a mucosal lining and an intact TM, a sound-conducting mechanism and the possibility of improvement in hearing and prevention of complications. 5 6

Various factors such as age, gender, smoking, pathology in the contralateral ear, size of the TM perforation and duration of the dry period have been reported 7 to affect the rates of success after tympanoplasty. Other iatrogenic factors supposedly associated with the surgical outcome of tympanoplasty are the type of procedure and approach chosen, as well as the surgeon's experience. 8 9 Similarly, myringosclerosis has been reported 8 9 10 as one of the possible surgical outcomes of tympanoplasty. Still, there are studies focusing only on the effect of myringosclerotic plaque removal on tympanoplasty outcomes. The current study aims to compare the outcomes of tympanoplasty in perforated TMs with and without myringosclerosis.

## Methods

The present prospective study was conducted in the Department of Otorhinolaryngology–Head and Neck Surgery of our institution over the course of 4 years. It was approved by the institutional Ethics Committee (IEC/60/2019), and it was conducted based on the Strengthening the Reporting of Observational Studies in Epidemiology (STROBE) statement. The study included 110 patients aged between 21 and 53 years, who had been diagnosed at the Outpatient Department with inactive mucosal COM, and in whom perforation alone or perforation with myringosclerosis had been found on the primary otoscopic examination.

The inclusion criteria were as follows: patients aged between 18 and 55 years; subjects diagnosed with inactive mucosal COM; and those with hearing loss < 45 dB. The exclusion criteria were patients under the age of 18 or over the age of 55; subjects diagnosed with active COM or squamosal COM; patients diagnosed with any temporal or intracranial complications of COM; and those with hearing loss > 45 dB.

Each subject was assessed for age; perforation size (small: affecting up to 25% of the TM; medium: affecting up to 50% of the TM; large: affecting up to 75% of the TM; and subtotal: affecting more than 75% of the TM); perforation location (quadrants); myringosclerosis, (site and size of the myringosclerotic plaques); and preoperative audiological findings (such as air conduction thresholds and air–bone gap for the frequencies of 500, 1,000, 2,000, and 4,000 Hz). The involvement of the mastoids was ruled out through bilateral oblique X-rays.

**Fig. 1:**
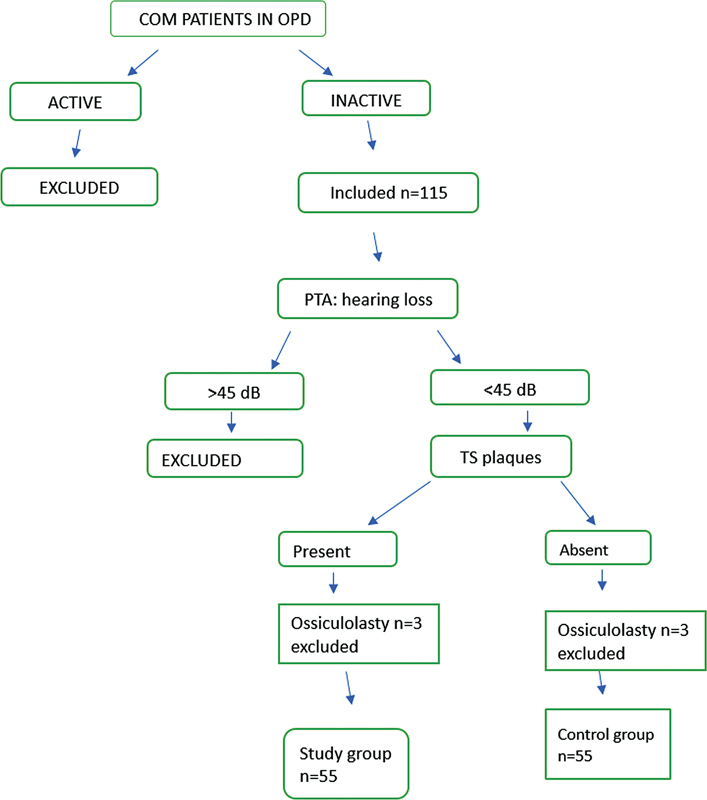
Patient selection in the Ear, Nose, and Throat Outpatient Department.

Thus, the sample divided into the case group, which included 57 subjects with TM perforation and myringosclerosis, and the control group, which included 58 subjects with TM perforation alone, without myringosclerosis. Both groups were matched in terms of age, gender, preoperative hearing thresholds and perforation (as shown in Fig. 1 ).

**Fig. 2:**
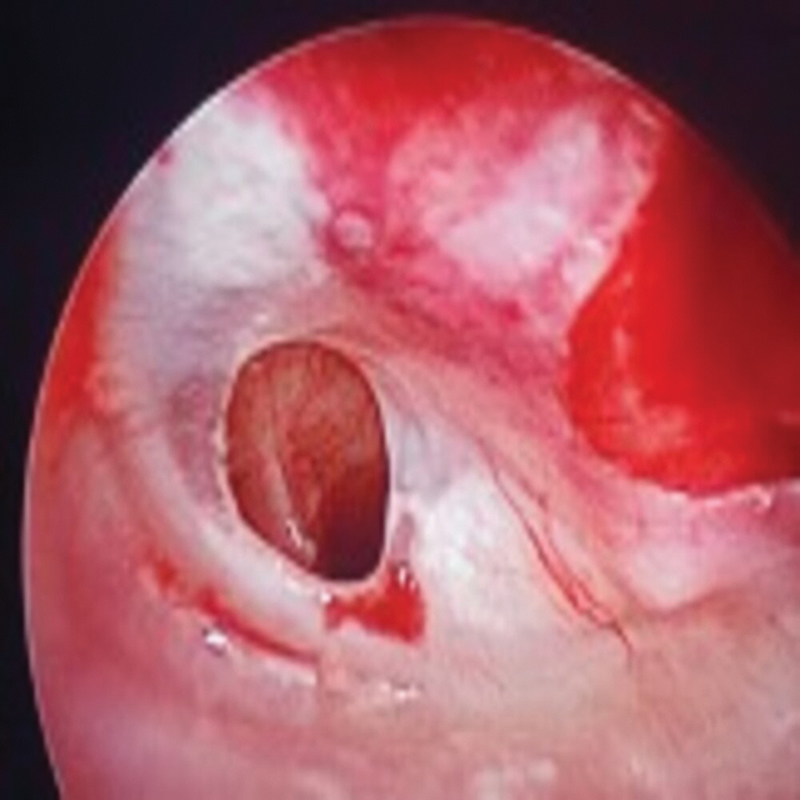
Intraoperative photograph showing a left tympanic membrane (TM) with perforation and myringosclerosis.

All primary tympanoplasty procedures were performed via the postauricular approach, with a temporalis fascia graft harvested and placed medial to the TM remnant, through the underlay technique. In the case group, myringosclerotic plaques were meticulously separated from the outer squamous layer of the TM and removed. Moreover, intraoperatively, three patients had tympanosclerosis of the ossicles, which was removed followed by ossiculoplasty if required; however, they were excluded from the study. In the control group, four patients had either ossicular necrosis or fixation of the ossicles, and they were managed through ossiculoplasty and excluded from the study as well ( Figs. 2 3 4 5 6 ).

**Fig. 3:**
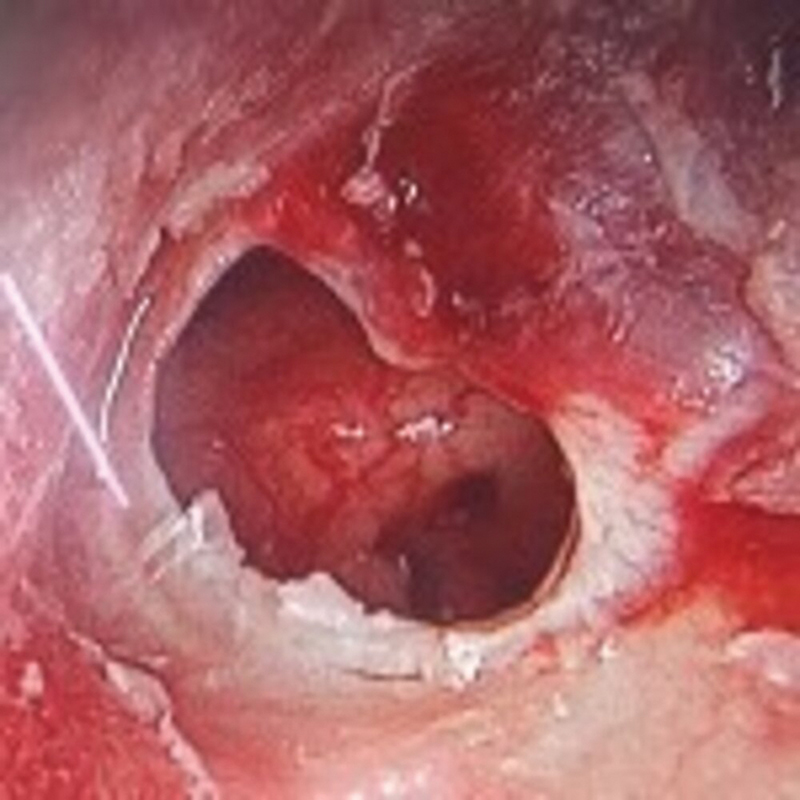
Intraoperative photograph showing a left TM with AIQ (Antero-Inferior Quadrant) and PIQ (Postero-Inferior Quadrant) perforation and myringosclerotic patch.

**Fig. 4:**
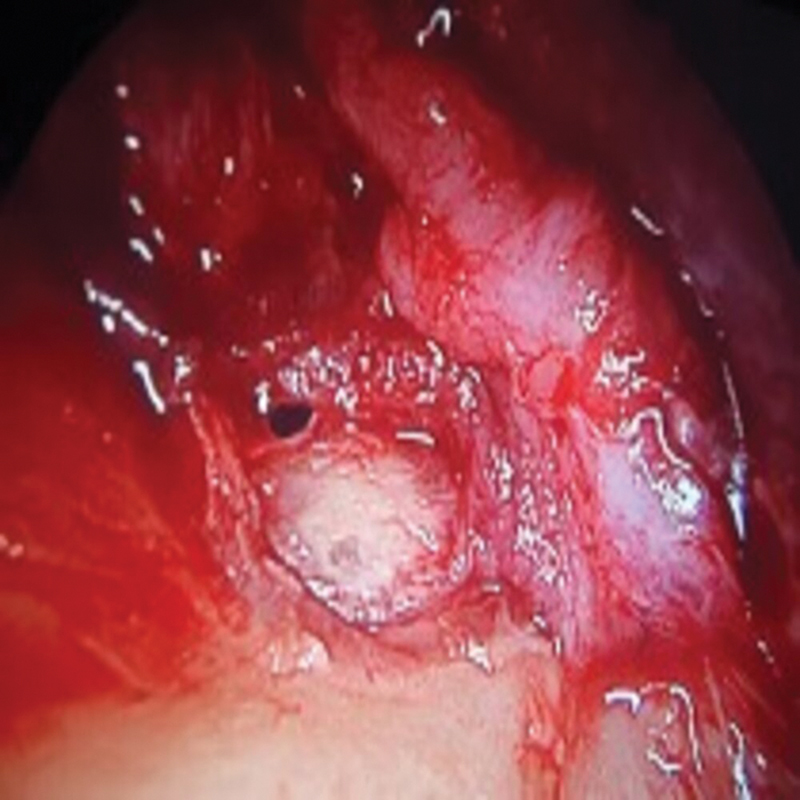
Intraoperative photograph showing the left side after the elevation of the tympanomeatal flap and the epithelial layer of the TM, exposing the myringosclerotic patch.

**Fig. 5:**
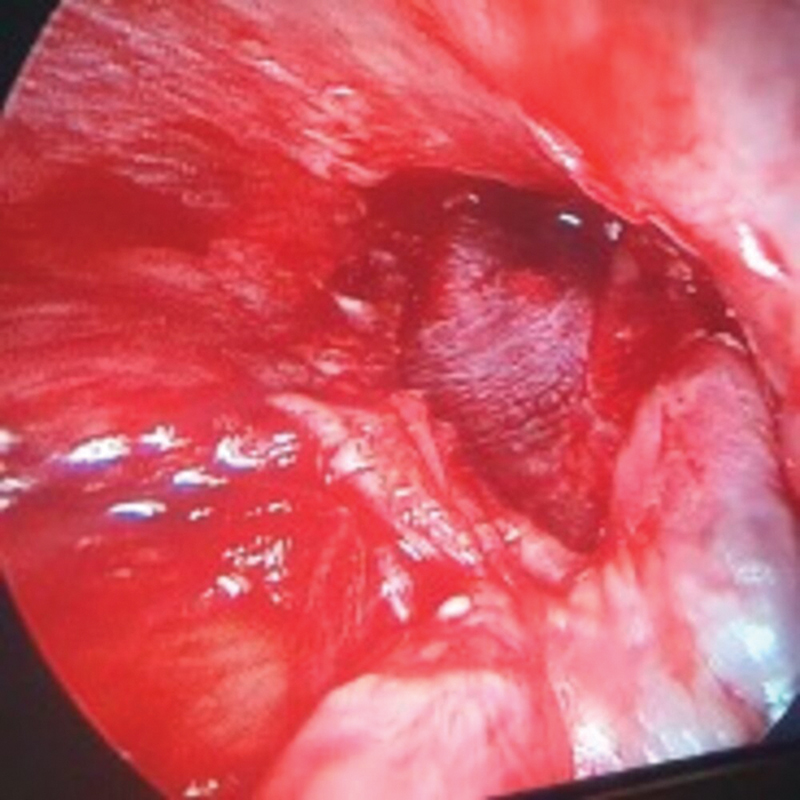
Intraoperative photograph showing the left side after graft placement and TM flap reposition.

**Fig. 6:**
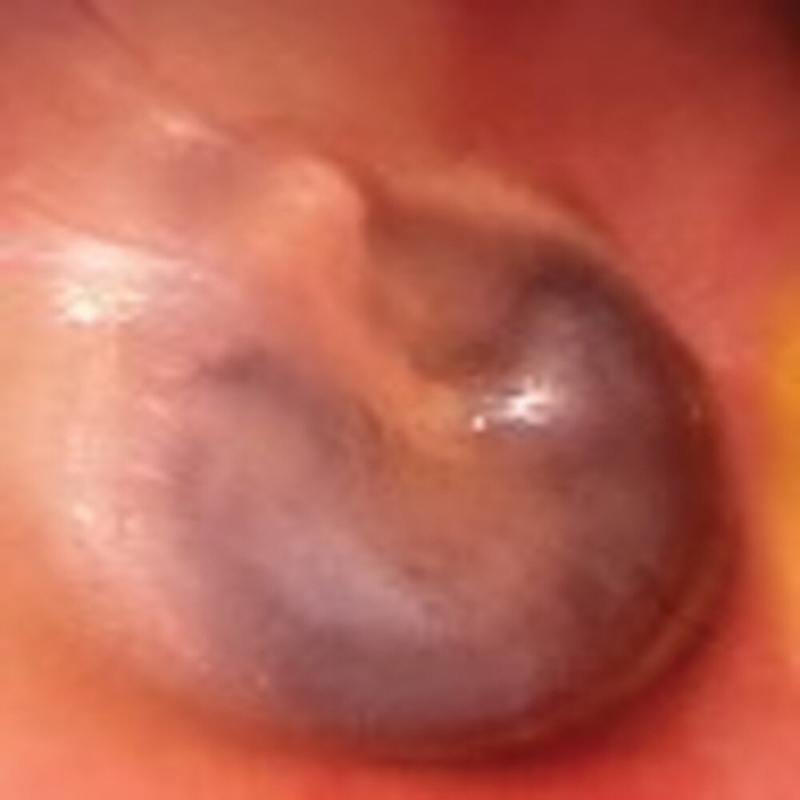
Postoperative photograph showing a right TM after 6 months of follow-up.

The patients were followed up for at least 6 months, and their audiological findings (air conduction thresholds and air–bone gap for the frequencies of 500, 1,000, 2,000, and 4,000 Hz, and speech reception threshold) were reviewed before and after surgery. We also assessed their hearing gain and surgical outcome (graft uptake, residual perforation or reperforation).

### Statistical Analysis

The data was entered into a Microsoft Excel (Microsoft Corp., Redmond, WA, United States) spreadsheet. Frequencies and percentages were used to express the categorical variables. Means and standard deviation values were used to express the continuous variables. The Fisher and Chi-squared tests were used to detect an association between the case group and the categorical variables. The two-sample *t* -test was used to detect the association between the case group and the continuous variables. Values of *p* < 0.05 were considered significant.

## Results

### Demographics

**Chart 1:**
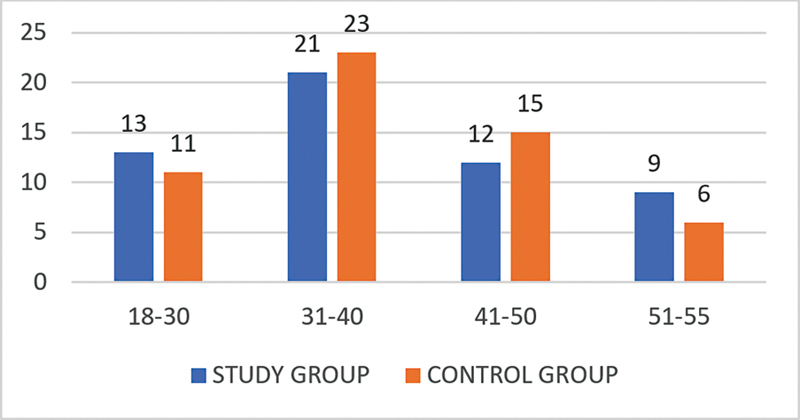
Age distribution showing that the difference between the cases and controls was not statistically significant.

In the present study, most subjects in both groups were in the aged between 31 and 40 years ( chart 1 ).

**Chart 2:**
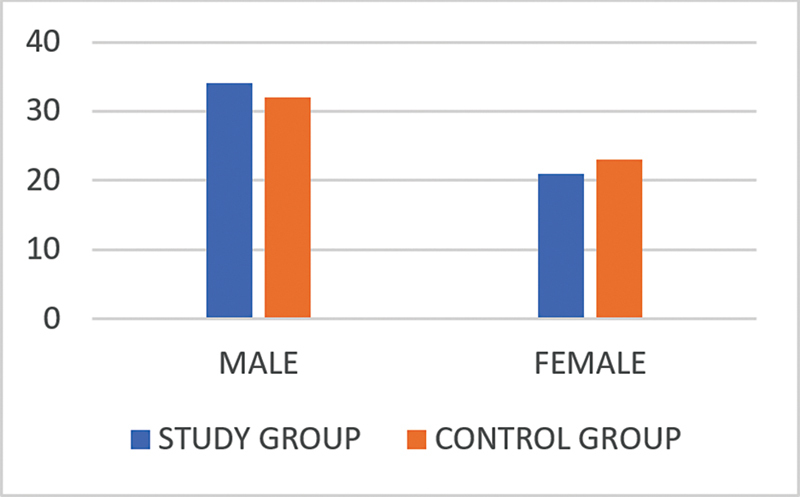
Gender distribution showing that the difference between the cases and controls was not statistically significant.

In the case group, there were 34 (61.8%) male and 21 (38.2%) female subjects, with a mean age of 35 ± 5.37 years and a male-to-female ratio of 1.6:1, while in the control group, there were 32 (58.2%) male and 23 (41.8%) female subjects, with a mean age of 37 ± 4.89 years and a male-to-female ratio of 1.4:1 ( chart 2 ). The difference between the cases and controls in terms of gender was not statistically significant ( *p* = 0.4).

### Clinical Presentation

The chief complaint in each group was a history of otorrhea ( Table 1 ).

**Table 1:** Clinical Presentation of the study sample

Clinical features	Case group (n)	Control group (n)
History of otorrhoea	55	55
Hearing loss	47	43
Tinnitus	11	10
Aural fullness	5	4
Vertigo/Dizziness	0	0
Itching	16	21

### Otoendoscopic Findings

#### Size of Tympanic Membrane Perforation

**Chart 3:**
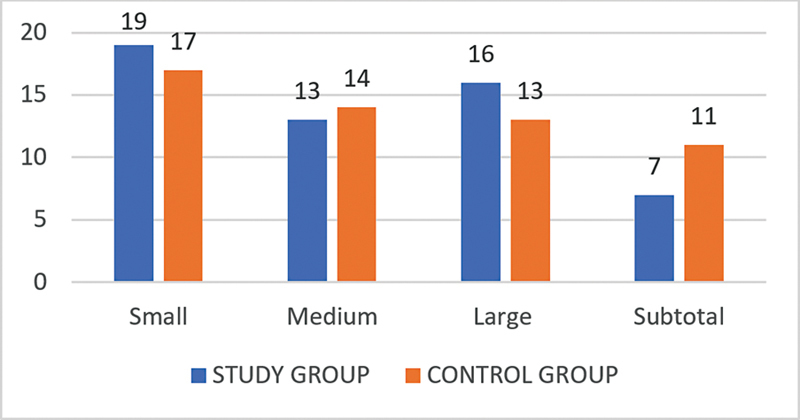
Distribution of on the study sample regarding the size of the TM perforation.

Regarding the size of the perforation, in the case group, it was small in 19 (34.54%) patients, medium in 13 (23.63%) subjects, large in 16 (29.09%) patients, and subtotal in 7 (12.7%) subjects. In contrast, in the control group, the perforation was small in 17 (30.1%) subjects, medium in 15 (27.27%) patients, large in 13 (23.6%) subjects, and subtotal in 10 (18.2%) patients ( chart 3 ). The difference between the cases and controls in terms of the size of the perforation was not statistically significant ( *p* > 0.05).

#### Site of Tympanic Membrane Perforation

**Chart 4:**
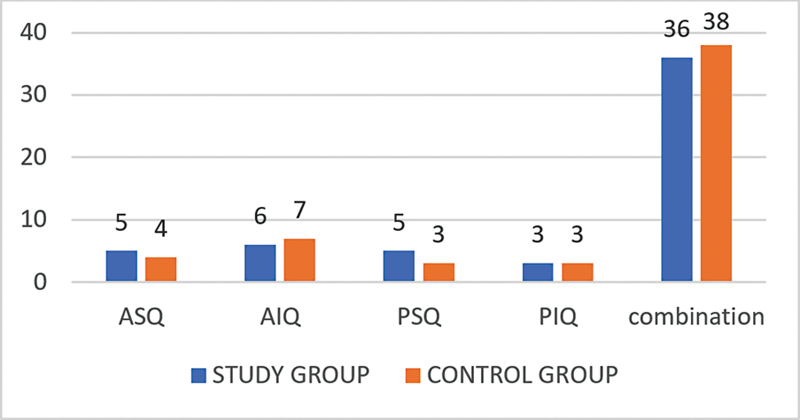
Distribution of the study sample regarding the site of the TM perforation.

As for the site of the perforation, in the case group, it was located in the anterosuperior quadrant in 5 (9%) patients, in the anteroinferior quadrant in 6 (10%) subjects, in the posterosuperior quadrant in 5 (9%) patients, in the posteroinferior quadrant in 3 (5.4%) subjects, and 36 (65.45%) patients presented partial involvement of the anterior and posterior quadrants. In the control group, the perforation was located in the anterosuperior quadrant in 4 (7.2%) patients, in the anteroinferior quadrant in 7 (12.7%) subjects, in the posterosuperior quadrant in 3 (5.4%) patients, in the posteroinferior quadrant in 3 (5.4%) subjects, and 38 (69.09%) patients presented partial involvement of the anterior and posterior quadrants ( chart 4 ). The difference between the cases and controls in terms of the site of TM perforation was not statistically significant ( *p* > 0.05).

#### Size of Myringosclerosis in the Case Group

**Chart 5:**
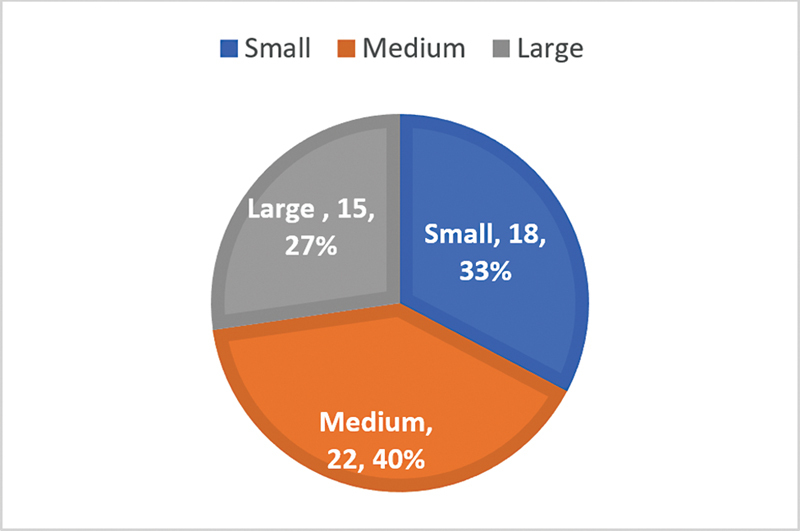
Size of the myringosclerosis in the case group.

Regarding the myringosclerotic patches, in the case group, they were small in 16 (29.1%) subjects, medium in 24 (43.7%) patients, and large in 15 (27.2%) subjects ( chart 5 ).

#### Site of Myringosclerosis in the Case Group

As for the site of the myringosclerosis, in the case group, it was located in the anterosuperior quadrant in 4 (7.3%) patients, in the anteroinferior quadrant in 2 (3.6%) subjects, in the posterosuperior quadrant in 7 (12.7%) patients, in the posteroinferior quadrant in 3 (5.4%) subjects, and 39 (70.1%) patients presented partial involvement of the anterior and posterior quadrants ( Table 2 ).

**Table 2:** Distribution of cases based on the site of myringosclerosis

Site of the myringosclerotic patch	Case group: n (%)
Anterosuperior quadrant	6 (10.9%)
Anteroinferior quadrant	2 (3.6%)
Posterosuperior quadrant	7 (12.7%)
Posteroinferior quadrant	3 (5.4%)
Parts of anterior and posterior quadrants	37 (67.27%)

#### Status of the Middle Ear Mucosa

Regarding the status of the middle ear mucosa, in the case group, it was congested and hypertrophied in 4 (7.3%) patients, congested and edematous in 14 (25.4%) subjects, and normal in 37 (67.3%) patients. In the control group, the middle ear mucosa was congested and hypertrophied in 7 (12.7%) patients, congested and edematous in 13 (23.6%) subjects, and normal in 35 (63.6%) patients ( Table 3 ). The difference between the cases and controls in terms of the status of the middle ear mucosa was not statistically significant ( *p* > 0.05).

**Table 3:** Distribution of cases based on the status of the middle ear mucosa

Middle ear mucosa	Case group: n (%)	Control group: n (%)	*p* -value
Congested and hypertrophied	4 (7.3%)	7 (12.7%)	0.17
Congested and oedematous	14 (25.4%)	13 (23.6%)	0.4
Normal	37 (67.3%)	35 (63.6%)	0.4

#### Preoperative Audiological Assessment

**Chart 6:**
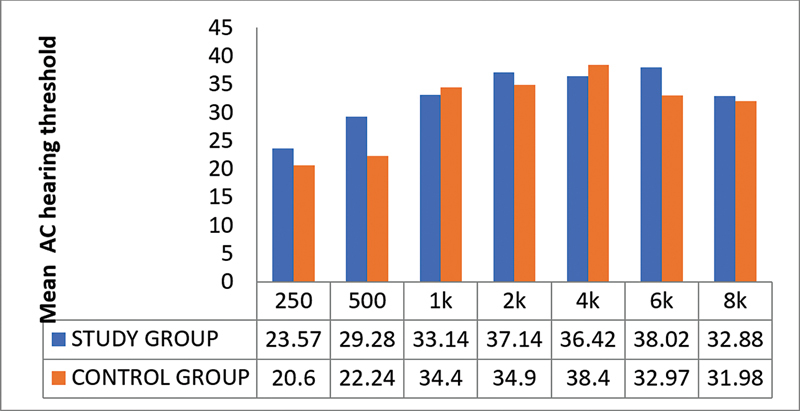
Distribution of the study sample according to the mean hearing threshold (air conduction) at different frequencies.

On the pure-tone audiometry, the differences in the mean values of the hearing thresholds of both groups at different frequencies were not statistically significant ( *p* > 0.05; chart 6 ).

#### Degree of Hearing Loss

The degree of hearing loss was assessed through the classification of the World Health Organization (WHO); the patients were categorized into 5 groups, and the difference between the cases and controls was not statistically significant ( *p* > 0.05; Table 4 ).

**Table 4:** Degree of hearing loss as per the classification of the World Health Organization

Degree of hearing loss (pure-tone average)	Case group: n (%)	Control group: n (%)	*p* -value
Mild (26–40 dB)	43 (78.2)	47 (85.5)	0.08
Moderate (41–55 dB)	12 (21.8)	8 (14.5)	0.3
Moderately severe (56–70 dB)	0	0	−
Severe (71–90 dB)	0	0	−
Profound (> 91 dB)	0	0	−

### Postoperative Status

Six months postoperatively, the graft uptake was successful in 91/110 (82.7%) patients in the sample: 49 (89.1%) in the case group and 42 (76.3%) in the control group; therefore, the outcome was better in the case group, but the difference between the cases and controls was not statistically significant ( *p* < 0.04; Table 5 ).. There were no granulations, or edema in the external auditory canal (EAC) in any of the patients at 6 months. Complete healing of the canal wall was present in all subjects. The improvement in hearing (measured in dB) is shown in Table 6 .

**Table 5:** Comparison of graft uptake between the case and control groups

Graft status	Case group: n (%)	Control group: n (%)	*p* -value
Uptake	49 (89.01)	42 (76.3)	0.04
Residual perforation	4 (7.2)	8 (14.5)	0.07
Reperforation	2 (3.6)	5 (9.01)	0.2

**Table 6:** Hearing gain in the study sample

Gain in pure-tone average (dB)	Case group: n (%)	Control group: n (%)
No gain	3 (5)	9 (16.3)
< 5	6 (10.9)	11 (20)
6–10	3 (5)	4 (7.2)
10–15	19 (34.5)	12 (21.8)
15–20	13 (23.6)	13 (23.6)
20–25	7 (12.7)	4 (7.2)
25–30	4 (7.2)	2 (3.6)

In the case group, 32 patients (58.1%) had perforation involving ≤ 50% of the TM and, in 23 subjects (41.8%) it involved > 50% of the TM. The rate of graft uptake was of 93.7% (30 patients) in perforations involving ≤ 50% of the TM, and of 82.6% (19 patients) in those involving > 50% of the TM. In the control group, 31 patients (56.3%) had perforation involving ≤ 50% of the TM and, in 24 subjects (43.6%), it involved > 50% of the TM. The rate of graft uptake was of 77.4% (24 patients) in perforations involving ≤ 50% of the TM, and of 75.1% (18 patients) in those involving > 50% of the TM ( Table 7 ).

**Table 7:** Comparison of graft uptake based on the size of the perforation

Perforation size			Graft uptake (%)	
	Case group: n	Control group: n	Case group: n (%)	Control group: n (%)
< 50%	32	31	30 (93.7)	24 (77.4)
> 50%	23	24	19 (82.6)	18 (75%)

### Comparison Between Pre- and Postoperative Hearing Loss

**Chart 7:**
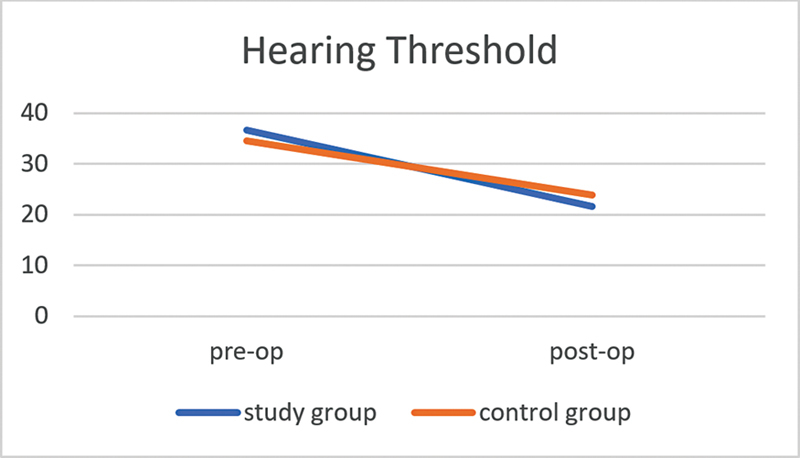
Line diagram showing a comparison of the pre- and postoperative hearing thresholds.

Regarding hearing loss, in the case group, the mean preoperative value was of 36.7 ± 7.9 dB, the mean postoperative value was of 21. 6 ± 6.3 dB, with a mean hearing gain of 15.1 ± 0.6 dB. In the control group, the mean preoperative value was of 34.6 ± 9.6 dB, and the mean postoperative value was of 21.9 ± 5.8 dB, with a hearing gain of 12.7 ± 0.9 dB. In comparison of both groups, the hearing thresholds showed a better outcome in the case group, but the difference between the cases and controls was not statistically significant ( *p* > 0.05; Table 8 and Chart 7 ).

**Table 8:** Comparison between pre- and post-operative air conduction threshholds (AC)

Air conduction threshholds (AC)	Case group (dB): mean ± standard deviation	Control group (dB): mean ± standard deviation	*p* -value
Preoperative AC	36.7 ± 7.9	34.6 ± 9.6	0.2
Postoperative AC	21.6 ± 6.3	21.9 ± 5.8	0.5
Hearing gain	15.1 ± 0.6	12.7 ± 0.9	0.07

## Discussion

Otitis media means inflammation of the middle ear cleft which includes the middle ear cavity, the ossicles and/or the mastoid antrum. It is an umbrella term for a spectrum of diseases, including acute otitis media (AOM), otitis media with effusion (OME/glue ear) and CSOM. A perforation is a sequela of any of the conditions. 1 2 3

A perforation can heal initially through the formation of scar tissue (myringosclerosis) with or without the development of a monomeric eardrum, in which the scar tissue extends into the middle ear (tympanosclerosis). In the middle ear, fibrocytes invade the lamina propria of the eardrum, leading to decomposition, hyalinization, and calcification, resulting in myringosclerotic plaques. 6 The hyalinisation and calcification are sequelae of chronic inflammation that can occur in any part of the TM and middle ear. 10

Myringosclerosis can occur simultaneously to a TM perforation. The success of tympanoplasty, a surgical procedure to repair the eardrum, depends on factors such as the patient's age, perforation size and location, involvement of the eardrum margins, and the condition of the other ear. 11 12 13 Adequate vascularization of the eardrum is critical for a successful tympanoplasty, as hyalinization and myringosclerosis can lead to poor blood supply, causing graft necrosis. Excision of sclerotic plaques may be necessary during surgery, which can enlarge the drum defect, requiring a larger graft to close it. 14

### Demographics

The present study, along with several others, 15 16 17 18 19 found no significant correlations involving patient age or sex and the success rate of eardrum closure or improvement in hearing after tympanoplasty. Even though age is considered a prognostic factor, some authors 20 21 22 suggest that the success rates of tympanoplasty in the pediatric population are slightly lower the rates in adults, possibly due to the higher rates of Eustachian tube dysfunction in children. However, there are conflicting opinions, with some studies 23 24 25 26 concluding that patient age does not impact the surgical outcomes of tympanoplasty.

### Effect of the Size of the Perforation

In the present study, the rate of graft uptake in the case group was of 93.7% (30 patients) in perforations ≤ 50% and of 82.6% (19 patients) in perforations > 50%. In the control group, it was of 77.4% (24 patients) in perforations ≤ 50% and of 75.1% (18 patients) in perforations > 50%. Muniraju et al. 27 reported that graft uptake was better in perforations ≤ 50%; however, this was not statistically significant. Onal et al., 7 Jain et al., 28 and Lee et al. 29 also reported significantly higher success rates with perforations < 50%. However, Wasson et al. 30 concluded that perforation size was not a statistically significant determinant factor for successful myringoplasty. The location and size of the perforation have been frequently examined in the literature, 25 29 30 and the subtotal perforations are more difficult to access for margins and to place grafts.

### Effect of the Location of the Perforation

In the present study, the location of the perforation did not affect graft uptake. Comparable to our results, many studies 31 32 33 34 35 have reported no influence of the site of the perforation on the surgical outcome after tympanoplasty. However, Pinar et al. 26 found that the graft success rate was higher for central perforations than for posterior and anterior perforations. Controversy remains regarding the influence of the location of the perforation on postoperative success. The location and size of the perforation have been frequently examined in the literature, 6 20 and the anterior perforations are more difficult to access and to place grafts, thereby leading to poor outcomes.

### Effect of Myringosclerosis

Myringosclerosis of the TM has been theorized 14 to cause poor feeding of graft material; in addition, the removal of sclerotic plaques during surgery results in a larger perforation.

Migirov and Volkov, 14 Pinar et al., 26 and Yurttafl et al. 34 reported that the absence of myringosclerosis increased the success rate of tympanoplasty. Onal et al., 7 van Stekelenburg and Aarts, 19 Muniraju et al., 27 and Wielinga et al. 36 found no correlation between myringosclerosis and the surgical outcome of tympanoplasty. However, in discordance with previous studies, 14 26 30 34 we found an overall graft success rate of 89.1% in the case group and of 76.4% in the control group.

The first layer of the TM, the epidermal layer, comprises epithelial skin cells filled with keratin, and it migrates outward in the centripetal direction from the umbo. To close the TM defect, cells at the edge of the wound must first loosen their adhesion to each other and to the basal lamina; this release enables epidermal cells to start migrating from the edge of TM, over the graft covering the defect. The denuding of the healed TM perforation edges and increased surface area of the raw margins created by the subepithelial excision of the sclerotic plaques aids the healing process, thereby increasing the rate of graft uptake. 36 37 38

## Limitations to the Study

The limitations to the present study include the small sample size, the fact that it was conducted in a single center, the short-term follow-up, and results that conflict with those of previous studies. 14 26 30 34

## Conclusion

To close the TM defect, cells at the edge of the wound must first loosen their adhesion to each other and to the basal lamina; this release enables epidermal cells to start migrating from the edge of TM, over the graft covering the defect. Appropriate denuding of the perforation edges, with the removal of sclerotic plaques, can lead to a high rate of surgical success regarding the closure of TM defects with coexisting myringosclerosis. This can be attributed to the increased surface area of the wound margins created by subepithelial excision of the MSP (Myringo-sclerotic patches).
